# Meticulous parade on naringin respecting its pharmacological activities and novel formulations

**DOI:** 10.22038/AJP.2022.20001

**Published:** 2022

**Authors:** Mahboob Alam, Farogh Ahsan, Tarique Mahmood, Arshiya Shamim, Saba Parveen, Mohammad Shariq, Vaseem Ahmad Ansari

**Affiliations:** *Department of Pharmacy, Faculty of Pharmacy, Integral University, Kursi Road, Lucknow, India*

**Keywords:** Naringin, Pharmacokinetics Pharmacological activity, Formulations, Flavonoids

## Abstract

**Objective:**

Medicinal plants having antioxidant potential possess numerous constituents which are responsible for different beneficial effects and are used as an alternative resource of medicine to lessen diseases linked with oxidative stress. Flavonoids are identified in the plants since ages and display wide spectrum of biological actions that might be able to stimulate the steps which are disturbed in different diseases. Flavonoids are significant natural compounds with various biologic properties, among which the most common is the anti-oxidant potential. Citrus flavonoids establish an important stream of flavonoids. Naringin, very common flavonoids present in the diet, belongs to the family of flavanone. It is the principal constituent of citrus family that contains flavonoids for example tomatoes, grapefruits and oranges.

**Materials and Methods:**

In this article, we reviewed naringin with respect to sources, chemical property, pharmacokinetics, pharmacological activity, and novel formulations. The literature survey has been done by searching different databases such as Psyc INFO, Science Direct, PubMed, EMBASE, Google, Google Scholar, Medline.

**Results:**

Naringin is known to behave as an antioxidant and possess anti-inflammatory, anti-apoptotic, anti-atherosclerotic, neuroprotective, anti-psychotic, anti-asthmatic, anti-diabetic, hepatoprotective, anti-tussive, cardioprotective, and anti-obesity activity. Further clinical studies using large sample sizes remain essential to obtain the appropriate dose and form of naringin for averting diseases. Furthermore, the therapeutic approach of these bioflavonoids is significantly inappropriate due to the lack of clinical evidence. Different plants must be explored further to find these bioflavonoids in them.

**Conclusion:**

The results of this exploration provides biological actions of bioflavonoid (naringin), predominantly on pharmacological and novel dosage forms of naringin.

## Introduction

Flavonoids are natural phenolic compounds with a broad range of bioactivity. Almost 4000 flavonoids, primarily from fruits, herbs, and vegetables, have been detected so far (Cook and Samman, 1996 ▶). The basic structure of the flavonoids consists of fifteen carbon atoms and 3 rings, 2 of which are benzene rings linked to a 3-carbon chain (Croft, 1998 ▶). Plants contain many forms of flavonoids, such as flavanone, flavone, polymethoxylated flavone, flavonol, and anthocyanin (Bhagwat et al., 2011 ▶; Ma et al., 2020 ▶).

Flavanones are major flavonoids which are very common in citrus fruits. In glycoside and aglycone forms, citrus flavanones are found. The major flavanone aglycones found in citrus fruits are eriodictyol hesperetin, naringenin, and isosakuranetin. In most citrus fruits, flavonone glycosides are more abundant than the flavanone aglycones (Bhagwat et al., 2011 ▶; Ma et al., 2020 ▶). Flavones are a flavonoid subgroup which, through a C2-C3 double bond, differ from flavanones. Flavones in citrus fruits are also found in glycoside and aglycone forms. In some plants like lemon and lime, a very small number of flavonols are present in the citrus fruits (Bhagwat et al., 2011 ▶; Ma et al., 2020 ▶).

Anthocyanins are water soluble pigments that are synthesized by phenylpropanoid pathway (Bhagwat et al., 2011 ▶; Ma et al., 2020 ▶). Such flavonoids are strong free radical scavengers and *in vivo*, they avoid oxidative stress (Croft, 1998 ▶; Ross and Kasum, 2002 ▶). Flavonoids are versatile in characteristics and chemical structure as a group of polyphenolic compounds. In fruits, vegetables, seeds, flowers, nuts and bark, they occur naturally and serve as major part of our diet (Middleton, 1993 ▶; Ratty and Das, 1988 ▶; Hackett, 1996 ▶). A broad range of biological effects have been mentioned as antibacterial, anti-inflammatory, anti-allergic (Hanasaki et al., 1994 ▶; Cook and Samman, 1996 ▶) and vasodilatory (Duarte et al., 1993 ▶) activities.

De Vry first observed naringin in the flower of grapefruit plant that grown in Java in 1857, but his results were not published at that time (Rangaswami et al., 1993 ▶). The name naringin is most likely derived from the word "Narangi" in Sanskrit, meaning "Orange" (Sinclair, 1972 ▶). As a bitter flavonone glycoside, naringin is a major active ingredient in citrus fruits, such as *Drynaria* *fortune*, *Citrus medica *L and *Citrus aurantium* L (Chen et al., 2016 ▶). Naringin is extracted from grapefruit (Citrus plant) (Papasani et al., 2014 ▶). Naringin is a bioflavonoid (flavanone glycoside) that gives grape fruit juice a bitter taste. In grapefruit, pummelo, sour orange, trifoliate orange, and kumquat, naringin is the chief constituents (Horowitz and Gentili, 1969 ▶; Alam et al., 2020 ▶). Various therapeutic eﬀects of naringenin (aglycone portion of naringin) such as cardioprotective (Arafa et al., 2005 ▶), cholesterol lowering, anti-Alzheimer's, nephroprotective, anti-aging, antihyperglycemic, anti-osteoporotic and gastroprotective (Jeon et al., 2004 ▶; Jagetia and Reddy, 2005 ▶), anti-inflammatory (Mohanty et al., 2020 ▶), antioxidant, anti-apoptotic, anti-carcinogenic, anti-osteoporotic and anti-ulcer activities have been studied (Wang et al., 2013 ▶; Rivoira et al., 2021 ▶).

For a long period of time, the plants containing naringin as a chemical constituent have been used as a natural source of medicine, and the use of plant compounds for pharmaceutical purposes has gradually increased in the world. The novelty of this manuscript justifies as, we collected the data related to characteristic, chemical constituents, pharmacological activities and novel formulations of naringin. Focus has been made on the pharmacological properties of naringin, so as to provide brief compiled information regarding its activities along with novel formulations as these formulations are hot topic for both clinicians and researchers. Nevertheless, the exact effects and mechanisms involved in pharmacological and toxicological effects of many of these chemicals remain to be cleared. This review aims to highlight the medicinal importance of bioflavonoid (naringin) and the journey of this folk ingredient to modern medicine.

## Materials and Methods

The searches were limited to the English language. The search was done from 1965-2020 and data were included from year 1969-2020. The scientific name of the plants was identified from the standard herbal literature. For collecting data, books, online materials, thesis, and scientific journals were also considered. The authors have utilized broad Major Exploded Subject Headings (MesH) terms and these keywords [Naringin, Naringin fruit] with the following suffix and prefix [pharmacological activity, phytochemistry, clinical trial, history, pharmacokinetics, chemical structure, species, morphological], using these words, the search was done using following search engines such as Psyc INFO, Science Direct, PubMed, EMBASE, Google, Google Scholar, Medline. Initially, articles were downloaded which were available as open-access files, the articles which were subscription-based were not downloaded, only the abstract was copied from such papers. Later the guideline of Systematic Reviews and Meta‐Analyses (PRISMA) was followed, the manuscripts which were not pertinent to the title of this paper were discarded. Summarizing the search strategy we have downloaded 106 manuscripts and noted the abstract of 23 papers. From which a total of 39 manuscripts were deleted as they were non-relevant, the remaining manuscripts were used for preparing this manuscript. All collected publications were reviewed manually by the third person to remove the chance of bias and also checked regarding the conflict of interest.

## Results

**Source**

Citrus fruits and Grapes contain a flavanone glycoside called naringin. It has a strong pungent taste for grapefruit juice (Jung et al., 2003 ▶; Kanaze et al., 2003 ▶). In grapefruit, pummelo, sour orange, trifoliate orange, and kumquat, naringin is also the primary bitter ingredient (Horowitz and Gentili, 1969 ▶). Naringin is a naringenin, an aglycone and neohesperidose flavanone glycoside bound to the -OH group at the carbon C-7 and it has a bitter taste (Braverman, 1949 ▶). When potassium hydroxide or another solid base is used, it is possible to make 1, 3-diphenylpropan-1-one from it, a compound that is 300–1800 folds sweeter as compared to sugar, with a refreshing sweet taste reminiscent of menthol (Jane, 2004 ▶). Naringin has a weak basic nature and is less soluble in water buffers (Tomasik, 2003 ▶). Rutinosc sugar molecule (L-rhamnose-D-glucose) is naringin and can be extracted with boiling mineral acid by hydrolysis. Naringenin is known as aglucose, which lacks bitter property of naringin. Although naringin is only moderately water-soluble (0.05% at 20°C), it might be crystallized when grapefruit is exposed to temperatures below freezing point (Hurst et al., 2018 ▶). Various *Citrus* species are listed in Table 1.

**Table 1:** Total content of naringin in different citrus fruits

**S.N.**	**Citrus species**	**Naringin content (mg/ml)**	**Reference **
1	*Citrus (C.) sinensis*	21.3	**(**Alam et al. 2014 ▶)
2	*Citrus bergamia*	22.3	
3	*Citrus clementina*	8	
4	*Citrus reticulate*	3383.6	
5	*Citrus paradise*	230	
6	*Citrus aurantium*	19.7	
7	Fruit juices/citrus juices (Naringin)	15.6	
8	Grapefruit, pure juice	30.8	
9	Pummelo, pure juice	84.8	
10	Orange (blond), pure juice	7	
11	Grapefruit (juice from concentrate)	37.8	
12	Pummelo hybrid/Grapefruit, pure juice	45.1	
13	Grapefruit, raw (color not specified)C. paradise (naringenin)	53.0	
14	Grapefruit, raw, white, all areas(*C. paradisi*) (naringenin)	21.3	
15	Grapefruit, raw, red and pink (*C. paradisi*) (naringenin)	32.6	
16	Grapefruit juice concentrate, frozen, white, unsweetened (naringenin)	31.2	
17	Grapefruit juice, canned, white, unsweetened (naringenin)	18.0	
18	Grapefruit juice, raw, white (naringenin)	18.2	

**Chemical property of naringin**

**Figure 1:**
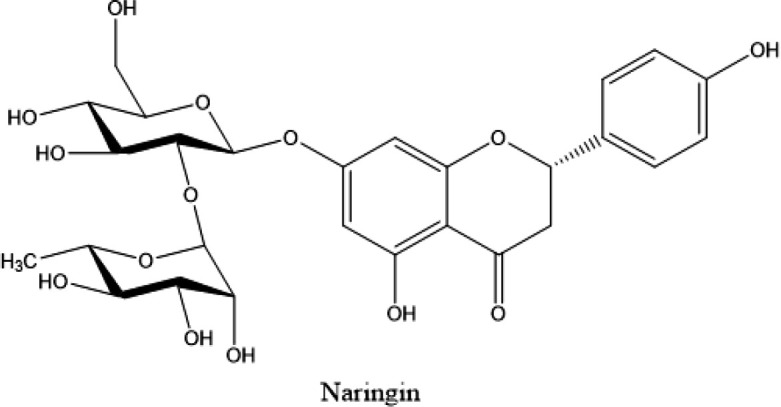
Chemical structure of naringin

Naringin is a flavanone glycoside obtained from citrus and grapes fruits (Figure 1) (with molecular formula C27H32O14 and 580.4 g/mol molecular weight). At the 7-carbon position, two rhamnose units are attached to its aglycon portion, naringenin. Naringin and naringenin are potent antioxidants (Renugadevi and Prabu, 2009 ▶; Jung et al., 2003 ▶). Compared to naringenin, naringin is very less potent as the sugar moiety induces steric obstruction by the scavenging community in the former. Naringin is slightly water soluble. Naringin is broken down into its aglycon naringenin in the intestine by the gut microflora; it is then absorbed from the gut (Choudhury et al., 1999 ▶). 

**Uses**

Natural flavonoids have unique physicochemical and physiological features that allow them to perform a broad array of functions. Naringin has a variety of biological and pharmacological properties that serve to minimize the risk of many diseases (Yin et al., 2015 ▶). Naringin is distinguished from other bioflavonoids by its bioactivity in the regulation of heart rhythm, its cardioprotective effect (Rani et al., 2013 ▶), and its ways to enhance lipid profile (Chanet et al., 2012 ▶), owing to its ability to lower LDL cholesterol and triglyceride levels in the blood, thereby increasing HDL cholesterol levels. All of this helps to keep blood pressure under control and prevent atherosclerosis from developing. Furthermore, it has been proven to have antidiabetic characteristics (Shen et al., 2018 ▶) and enhance thermogenesis (Silver et al., 2011 ▶), making it an ideal element in dietary supplement formulations aimed at promoting health and weight loss.

**Pharmacokinetics**

The oral absorption of naringin, obtained from citrus fruits indicated that it was not well absorbed in the gastrointestinal tract (GIT) in its original form (Kanaze et al., 2004 ▶). Naringin free form was partially present in plasma in rats and humans, and the predominant metabolite was naringenin glucuronide (Felgines et al., 2000 ▶; Fang et al., 2006 ▶). Naringin was rapidly absorbed into the serum by entering the first concentration peak at 15 min and another at 3 hr oral dose of naringin monomer. It can not be observed after 480 min of dosing, which could be due to rapid metabolism (5.075 mg/kg). The area under the curve (AUC_all_) and mean residence time (MRT) were 274.8070 min, mg/L and 114.0243 min (Li et al., 2013 ▶).

Naringin can also influence the absorption, metabolism or removal process of candesartan (CDS), thereby affecting the absorption process in the intestine (Surampalli et al., 2015 ▶). It is suggested that the absorption of diterpenoid (oridonin) in rats is much greater than that of flavonoid glycoside (naringin) (Jin et al., 2015 ▶). Naringin was administered by duodenal cannula. The mean naringin Cmax occurred at 18.8±3.8 min in portal plasma (determined to achieve tmax in portal plasma) and was significantly greater than in mesenteric lymph fluid. The Cmax of naringin was approximately 1.7-fold higher in bile than in jugular plasma (Tsai and Tsai, 2012 ▶). The total absorption of naringin in rats and dogs was found to be 44.1% and 34.4%, respectively. It has also been reported that the pharmacokinetic evaluation of naringin were significantly altered by administration of high fat diet (Bai et al., 2020 ▶; Joshi et al., 2018 ▶).

**Pharmacological activity**

Naringin possesses potential pharmacological properties. The literature suggests that naringin has mainly antioxidant potential which provides a bridge between traditional medicine and western medicine due to its pharmacological potential. The literature available on these properties has been summarized below:

**Anti-inflammatory activity**

The anti-inflammatory effect of naringin in cisplatin-induced renal injury in rats has been studied. The administration of naringin on various doses (20, 50 or 100 mg/kg) has shown to protect against impaired renal function, eliminate the decrease in antioxidant enzymes and suppress increases in thiobarbituric acid reactive substances (TBARS), tumor necrosis factor (TNF-α) concentrations and nitrite (Chtourou et al., 2016 ▶). According to another study, naringin is an important anti-inflammatory agent in cisplatin-induced rats to attenuate chronic pulmonary neutrophilic inflammation (Nie et al., 2012 ▶). Anti-inflammatory effect of naringin in cigarette smoke-induced chronic bronchitis in guinea pig suggested that naringin may have novel therapeutic potential for chronic bronchitis treatment (Luo et al., 2012 ▶). Naringin inhibited the increased expression of TNF-α and high mobility group box protein 1 (HMGB-1). It was shown that oral naringin treatment may be beneficial in the treatment of patients with rheumatoid arthritis (Kawaguchi et al., 2011 ▶).

**Anti-oxidant activity**

Effects of hesperidin and naringin on antioxidant activity and plasma lipid profile in rats fed on diet containing cholesterol displayed that the bioactive citrus fruit compounds are effective reducers of plasma lipids and possess potential antioxidant activity (Gorinstein et al., 2007 ▶).

The assessment of antioxidant effects of naringin and probucol was reported by Jeon et al. In the antioxidant protection system, probucol was very potent, while naringin showed a comparable antioxidant potential based on increasing gene expression in antioxidant enzymes (Jeon et al., 2002 ▶). Results suggest that it was possible to defend against kidney disease at different doses of naringin, to eliminate the decrease in antioxidant enzyme activity and to suppress the rise in TBARS, nitrite, TNF alpha concentrations, and to enhance the histological changes caused by cisplatin (Chtourou et al., 2016 ▶). It also lowered the oxidative biomarkers malondialdehyde (MDA) and lactate dehydrogenase (LDH) and increased the levels of catalase (CAT) and superoxide dismutase (SOD) (Papasani et al., 2014 ▶).

**Cancer**

The *in vivo* action of naringin in Walker 256 carcinosarcoma bearing rats has been studied. Furthermore, complete tumor regression was present in 2 rats. Inhibition of tumor development, decreased IL-6 levels, TNF-alpha and augmented survival in Walker 256 carcinosarcoma-bearing rats treated with naringin strongly showed that it has anticarcinogenic potential (Camargo et al., 2012 ▶). Naringin treatment prevents lipid peroxidation and liver damage, proving the antioxidant protection mechanism in rats after diethylnitrosamine (DEN)-induced liver carcinogenesis (Thangavel et al., 2012 ▶).

**Anti-tussive effect**

The anti-tussive activity of naringin on electrical stimulation-induced cough in guinea pigs was recorded. Naringin is not a primary antitussive drug, either the sensory neuropeptide mechanism or the activation of ATP-sensitive K^+^ channels does not have a peripheral antitussive activity (Gao et al., 2011 ▶).

**Anti-asthmatic effect**

The anti-asthmatic activity of naringin was estimated. In this experiment the ovalbumin caused inflammation of the airways in the mouse. Flow cytometry experiments found that Th2 cells and enhanced Th1 cells were significantly inhibited by naringin. Naringin increased T-bet and inhibited GABA3 significantly (Guihua et al., 2016 ▶).

**Cardiovascular disorders**

Naringin inhibited lipopolysaccharide induced increased activity of TNF-alpha, IL-1β and IL-6 to relieve the inflammatory action in the heart. Additionally, supplementation of naringin significantly elevates SOD levels and prevents oxidative stress parameter compared to lipopolysaccharide-induced injury. Finally, treatment with naringin potentially reduced the expression of pro and anti-apoptotic (BAX and BCL-2) respectively in cardiac tissue (Xianchu et al., 2016 ▶). Naringin was shown to have anti-lipoperoxidative and antioxidant function in cardiac toxicity that was experimentally induced (Rajadurai and Prince, 2006 ▶). 

**Antiatherogenic effects**

Naringin significantly decreased the development of fatty streaks and filtration of neointimal macrophages, it also inhibited the activity of intercellular adhesion molecule 1 (ICAM-1) in endothelial cells and has hepatoprotective role unlike lovastatin (Choe et al., 2001 ▶). Naringin decreased plasma non-HDL level and also endothelial dysfunction biomarkers, showing its protective impact (Chanet et al., 2012 ▶).

**Anti-hypertensive effect**

By downregulating the inflammatory markers including TNF-alpha and IL-β, naringin has prevented hypertension and ocular dysfunction. Naringin can be a useful medication to mitigate apoptosis, inflammatory markers and metabolic nucleotide disorders in hypertensive rats through the NOS/cGMP/PKG signaling pathways for identifying myocardial damage (Akintunde et al., 2020 ▶). Anti-hypertensive effect of naringin in renal artery occlusion induced hypertension in rats, showed altered left ventricular function at different time intervals after the clamp was removed. The study concluded that treatment with naringin has markedly altered SOD, MDA and GSH levels (Visnagri et al., 2015 ▶).

**Anti-diabetic effect**

When treated with naringin, the level of plasma cholesterol and triglyceride were reduced from 84.84±1.62 to 55.59±1.50 mg/dl and 123.03±15.11 to 55.00±0.86 mg/dl respectively (Rotimi et al., 2018 ▶). When naringin is administered to hyperglycaemic rats, there is significant decrease in glucose levels, an increase in insulin levels, a decrease in TBARS and H_2_O_2_ levels, and increase in overall antioxidant activity with increase in the activity of the antioxidant enzyme (CAT, GPx, SOD and paraoxonase) (Mamdouh and Monira, 2004 ▶).

**Nephroprotective effect**

The results of Singh and Chopra, 2004 has demosntrated that ROS play a degrative effect in renal injury caused by ischemia/reperfusion (I/R) and naringin probably exerts renoprotective effects via antioxidant and radical scavenging activities (Singh and Chopra, 2004 ▶). Histopathological and other studies revealed that the molecular and biochemical findings are relevant nephroprotective effect of naringin in cisplatin caused nephrotoxicity in rats (Abd Elmonem et al., 2018 ▶).

**Hepatoprotective effect**

The increase in Fas/FasL/caspase-3 protein expression and in the Bax/Bcl-2 ratio have shown that diabetes increased both pathways of apoptosis, effects which were abrogated by naringin treatment (Rodríguez et al., 2018 ▶). The hepatoprotective effects of naringin was evaluated in carbon tetrachloride (0.5 ml/kg; subcutaneous) induced liver damage in rats. The altered changes were significantly restored in rats pretreated with naringin (Badr et al., 2009 ▶).

**Ulcer**

The gastroprotective effect of naringin in rats with ethanol caused gastric-lesions has been studied. Naringin has been shown to have cytoprotective action toward ethanol injury in rats, however this property seems to be mediated by a non-prostaglandin dependent mechanisms (Martin et al., 1994 ▶).

**Wound healing**

Evaluation of the wound healing action of naringin ointment formulation (NOF) in experimental wound models showed a substantial decrease (p<0.05) in the wound surface area and in the epithelial duration, while the wound contraction rate significantly increased (p<0.05). Histological changes in wound skin were also restored by the NOF (Kandhare et al., 2016 ▶).

**Neuroprotective effect**

Neuroprotective activity of naringin in streptozotocin induced painful diabetic neuropathy in rats has been evaluated. The result suggest that chronic naringin therapy reduced the nociceptive threshold level, membrane-bound inorganic phosphate enzyme, endogenous antioxidant oxidative-nitrosative stress, neural cells apoptosis and inflammatory mediators. (Kandhare et al., 2012 ▶). Naringin therapy resulted in substantial reduction in attenuated oxidative damage and cognitive function as proved by lower levels of MDA and nitrite and decreased levels of glutathione and acetylcholinesterase compared with control levels (Kumar et al., 2010 ▶). The study demonstrates that by attenuating hyperammonemia, naringin effectively decreased neurotoxicity, indicating that it possess neuroprotective properties (Ramakrishnan et al., 2016 ▶). Neuroprotective activity of naringin in streptozotocin induced diabetic in rats significantly reduced MDA levels, elevated SOD levels and also increased TNF-α, IL-1β, and IL-6. PPARγ expression was also increased when pretreated with naringin (Liu et al., 2016 ▶).

**Table 2:** The summary of pharmacological activities of naringin

**Type of study**	**Subject**	**Dose/Route**	**Finding & Inferences**	**Reference**
Anti-inflammatory	Rat	20, 50 or100 mg/kg; per oral	Naringin can be a useful dietary supplement for reducing the risk of nephrotoxicity caused by anticancer drugs like cisplatin in cancer chemotherapy. Cisplatin-induced renal dysfunction can be mitigated with naringin supplementation. During cisplatin toxicity, naringin was able to restore redox equilibrium, suppressing inflammation, NF-kB activation, and apoptosis.	(Chtourou et al. 2016 ▶)
	Rat	20, 40 & 80 mg/kg; intragastric	Naringin dependently decreased cigarette smoke, caused inflammatory cell invasion, bronchial wall thickening, and average alveolar airspace expansion.	(Nie et al. 2012 ▶)
	Guinea pig	9.2, 18.4 and 36.8 mg/kg; per oral	Naringin effectively reduced exposure to chronic smoke-induced enhanced cough, inflammation of the airways, AHR and suppressed the decline in SOD activity and LXA4 airway content in this guinea pig model.	(Luo et al. 2012 ▶)
	Mice	150 mg/kg/0.3 ml; per oral	Oral administration of Naringin to mice by collagen-induced arthritis reduced the severity of clinical symptoms in knee joints.	(Awaguchi et al. 2011)
Anti-oxidant activity	Wistar rats	0.46–0.92 mg in 1 to 2 ml of water; per oral	The rise in plasma-lipid levels induced by cholesterol feeding was significantly reduced by diets supplemented with naringin.	(Gorinstein et al. 2007 ▶)
	Male rabbit	Naringin- 0.5 gr/kg Probucol- 0.5 gr/kg; per oral	Naringin showed a comparable antioxidant ability by increasing gene expression followed by overexpression of antioxidant enzymes.	(Jeon et al. 2002 ▶)
Anti-apoptotic effect	Rat	20, 50 or 100 mg/kg; per oral	Naringin protected kidney function, reversed the decrease in the activity of antioxidant enzymes, and suppress increases in nitrite, TNF-α and TBARS levels.	(Chtourou et al. 2016 ▶)
Carcinogenesis	Rat	10, 25 and 35 mg/kg; i.p.	Naringin, prolonged the tumor growth increased the survival rate and avoided cachexia. Naringin can be used as a potent antitumor agent as highlighted in these findings.	(Camargo CA et al. 2012 ▶)
	Rat	40 mg/kg; per oral	Treatment with naringin prevents lipid peroxidation, liver damage & protects the antioxidant protection mechanism from liver carcinogenesis.	(Thangavel et al. 2012 ▶)
Anti-tussive effect	Guinea pig	15, 30, and 60 mg/kg; i.v.	Naringin possess the anti-tussive effect probably by suppressing the cough center of the brain.	(Gao et al. 2011 ▶)
Anti-asthmatic	Mouse	5 mg/kg and 10 mg/kg; per oral	Naringin possess anti-asthmatic effect by inhibiting IL-4, improved IFN-γ, and suppressed both the formation of eosinophils and mucus overproduction in mice with OVA-induced asthma.	(Guihua et al. 2016 ▶)
Myocardial effect	Mouse	100 mg/kg; per oral	Naringin has cardioprotective effects by controlling inflammatory response, oxidative stress, and apoptotic reaction.	(Xianchu et al. 2016 ▶)
	Rat	10, 20 and 40 mg/kg; per oral	The biochemical and histopathological results obtained from the research indicate that naringin provides myocardium defense against oxidative stress induced by ISO in rats.	(Rajadurai and Prince, 2006 ▶)
	Male albino rats	100 & 200 mg/kg; per oral	The naringin showed protective effects against myocardial injury. The treatment with naringin significantly reduced the development of free radicals, the generation of lipid peroxides and the leakage of cytosolic enzymes, characterized by decreased biomarker levels.	(Papasani et al. 2014 ▶)
Antiatherogenic effect Antiatherogenic effect	Rabbit	500 mg/kg; per oral	Naringin, significantly reduced the development of fatty streaks and neo-intimal macrophages in filtration and suppressed the activation of ICAM-1 in endothelial cells. It also has a hepatoprotective effect.	(Choe et al. 2001 ▶)
	Mice	High-fat/High cholesterol diet (−41%); per oral	The antiatherogenic effect of naringin displayed nutritionally achievable dose supplemented specifically for diet-induced atherosclerosis.	(Chanet et al. 2012 ▶)
Anti-hypertensive Anti-hypertensive	Rat	80 mg/kg; per oral	Results suggest that naringin can be used as an antihypertensive agent	(Akintunde JK et al. 2020 ▶)
	Rat	20, 40 and 80 mg/kg; per oral	Naringin, through its antioxidant activity, exerts antihypertensive potential.	(Visnagri A et al. 2015 ▶)
Anti-diabetic	Rat	50, 100 and 200 mg/kg; per oral	Naringin could reverse T2DM-associated atherosclerosis by reducing dyslipidemia through HDL-mediated reverse cholesterol transport and protecting lipoprotein from oxidation by raising paraoxonase activity.	(Rotimi et al. 2018 ▶)
	Rat	0, 10, 20, 40, or 80 mg/kg; i.p	Multiple doses of Naringin significantly improved the hypoglycemic & antioxidant activity of diabetic rats caused by streptozotocin.	(Mamdouh and Monira, 2004 ▶)
Nephroprotective effect	Rat	400 mg/kg; per oral	The results suggest that the renal injury induced by I/R relates to its capacity to produce free radicals and that the ability of naringin to defend against this injury is possibly due to the improvement of this drug's antioxidant potential & free radical scavenging activity.	(Singh and Chopra, 2004 ▶)
	Rat	80 mg/kg; per oral	Naringin has shown a strong protective effect against cisplatin-caused nephrotoxicity through its antioxidant, anti-inflammatory and apoptotic activities.	(Abd Elmonem et al. 2018 ▶)
Hepatoprotective effect	Rat	40 mg/kg; Subcutaneous	Naringin preserves the liver from the damage caused by streptozotocin-induced diabetes and may be a novel clinical technique for the prevention of type-1 diabetes mellitus-related non-alcoholic liver fat disease.	(Rodríguez et al. 2018 ▶)
	Rat	300 mg/kg; per oral	Naringin exerts a preventive effect, likely through its antioxidant action, on CCl_4_-induced haematology and liver damage in rats. Thus the, supplementation therapy with naringin can effective in reducing tissue damage in patients exposed to toxic doses of CCl_4_.	(Badr et al. 2009 ▶)
Gastroprotective effect	Rat	400 mg/kg; per oral	Naringin showed a cytoprotective function against ethanol damage, but this effect tends to be mediated by pathways other than prostaglandins.	(Martin et al. 1994 ▶)
Wound healing	Rat	Topical	Naringin exerts its wound healing ability through the down-regulating expression of the inflammatory & apoptotic mediators while the up-regulating expression of the growth factors, thereby modulating the appearance of the collagen-1 gene to induce angiogenesis leading to wound healing.	(Kandhare et al. 2016 ▶)
Neuroprotective effect	Mice	2.5, 5 and 10 mg/kg; i.p.	Naringin induced anxiolytic-like activity in mice and improved cognitive performance. Naringin substantially increased the activity of SOD, CAT, and GSH concentration and decreased nitrite levels and MDA and brain acetylcholinesterase activity.	(Ben-Azu et al. 2019 ▶)
	Mice	50 and 100 mg/kg; i.p.	Oxidative damage, neurobehavioral alterations and recovered mitochondrial enzyme complex actions were significantly attenuated when treated with naringin showing recovery from depression.	(Aggarwal et al. 2010 ▶)
	Rat	100 mg/kg; per oral	Naringin possesses a strong add-on therapeutic activity against schizophrenia caused by ketamine.	(George et al. 2020 ▶)
	Rat	20, 40 & 80 mg/kg; per oral	Naringin exhibit its neuroprotective impact by downregulation of free radical, cytokine including TNF-α thus preventing diabetes-induced neuropathic pain over modulation of endogenous biomarkers.	(Kandhare et al. 2012 ▶)
	Rat	40 and 80 mg/kg; per oral	Naringin's multiple effects firmly support its neuroprotective effects toward colchicine-induced cognitive impairment and oxidative injury.	(Kumar et al. 2010 ▶)
	Rat	80 mg/k; per oral	The results indicate that naringin exerts protective efficiency related to neuronal complications against hyperammonemic rats induced by NH_4_Cl.	(Ramakrishnan et al. 2016 ▶)
	Rat	100 mg/kg; per oral	The study has shown that Naringin may be an effective drug to enhance learning and memory efficiency in DACD.	(Liu et al. 2016 ▶)

NF-kB: Nuclear Factor kappa-light-chain-enhancer of activated B cells; AHR: Airway hyperresponsiveness; SOD: Superoxidase dismutase; LXA4: lipoxin A4; TBARS: Thiobarbituric acid reactive substances; TNF-α: Tumour necrosis factor α; IL-4: Interleukin 4; IFN-γ: Interferon gamma; OVA: Ovalbumin; ISO: Isoprenaline; ICAM-1: intercellular adhesion molecule 1; T2DM: Type 2 diabetes mellitus; HDL: high-density lipoprotein; CCl4: carbon tetrachloride; CAT: catalase; GSH: reduced glutathione; MDA: Malondialdehyde; NH4Cl: Ammonium chloride; DACD: Diabetes-associated cognitive decline

**Table 3:** Novel formulations of naringin

**Purpose of study**	**Formulation approach**	**Objective**	**Method of preparation**	**Result**	**Reference**
To prepare deformable liposomes of Naringin for improved anti-inflammatory activity	Deformable liposomes	For anti-inflammatory skin activity deformable liposomes of Naringin was made	Thin- film hydration technique	When compared to marketed preparation, the liposomes showed increased anti-inflammatory activity in an in-vitro assay	(Pleguezuelos-Villa et al. 2018 ▶)
To prevent development of resistance toward chemotherapeutic agents by combining Naringin and paclitaxel	Mixed micelles	To develop anticancer medicine with combining paclitaxel and Naringin	Solvent diffusion method	Naringin synergistically increased its intracellular intake and 65 % *in-vitro* cytotoxicity	(Jabri et al. 2019 ▶)
To develop formulation which may prevent Naringin release bursting and osteogenesis	Microspheres	To prepare Naringin-loaded microsphere/sucrose acetate isobutyrate hybrid depots and improve osteogenesis	Single-nozzle-electro-spraying setup	Microspheres showed effective biocompatibility and osteogenic potential in-vitro. Ng-m-SAIB may demonstrate promising for bone repair to be a sustained release carrier	(Yang et al. 2019 ▶)
To incorporate into sunscreen creams which may increase protection against U.V. radiation	Ethosomes of Naringin	To improve the penetrating capacity and retention capacity of Naringin into sunscreen creams	Hot method and mechanical dispersion method	Ethosomes showed a pronounced skin penetration for Naringin across the skin and had a good skin retention and U.V. protection ability	(Gollavilli et al. 2020 ▶)
To prepare a dosage form in form of nano-capsule which have good bioavailability, bioavailability, biotransformation and distribution	Naringin-loaded Nano-capsules	To formulate nano-capsules of Naringin and to evaluate the toxicity	Interfacial- deformation technique	The ethosomes showed desired pharmacokinetic effect and there was no indication of toxicity by nano-capsules	(Budel et al. 2020 ▶)
To prepare a gum tragacanth stabilized green nanoparticles for increasing bactericidal activity	Naringin nanoparticles	To formulate green gold gum tragacanth loaded Naringin nanoparticles	Through magnetic stirring the color change was observed	Naringin's bactericidal potential was increased when it was loaded into AuNPs against different bacterial strains	(Rao et al. 2017 ▶)
To prepare a dosage form with increased drug release	Ternary nanoparticles containing amylose, alpha-linoleic acid, and beta-lactoglobulin complexed with Naringin	To formulate Naringin-nanoparticle inclusion complex for increased bio accessibility and thereby bioavailability	Through magnetic stirring the preparation of ternary nanoparticles and inclusion complex with Naringin was prepared	Naringin gradually released from the complex mixture and nanoparticles are promising carrier for increased bioavailability of Naringin	(Feng et al. 2017 ▶)
To prepare high catalytic properties of alpha-amalyse	Enzyme immobilized in magnetic nanoparticles of Naringin.	To formulate alpha-amalyse immobilized functionalized Magnetic NPs	Magnetite nanoparticles followed by immobilization of alpha-amalyse onto magnetic nanoparticle containing Naringin	Improvement in enzyme catalytic properties made nano-biocatalyst a good candidate in bio industrial applications	(Defaei et al. 2018 ▶)
To prepare a formulation having better anti-tumor activity of Naringin against hepatocellular carcinoma	Nanostructured lipid carrier with Naringin & coix seed oil.	To develop a Nanostructured lipid carrier containing Naringin and coix seed oil for the treatment of hepatocellular carcinoma	Ultrasonic- melt emulsification method.	The drug release and synergistic antitumor effect provides new insight against cancer	(Zhu et al. 2020 ▶)
To develop sustainable agriculture by using Naringin novel formulation	Naringin & citric acid in polycaprolactone microcapsules	Plant development and sustainable agriculture with polycaprolactone microcapsules containing Naringin & citric acid	Combination of a double emulsion method of water-in-oil-in-water and a solvent evaporation technique	The use of PCL 45000 Mw for the synthesis of MCs containing citric acid or Naringin may be a viable alternative to the current need for environmentally friendly agricultural practices. MCs containing Naringin have a 30-day slow release that is unaffected by pH, indicating that it should be used in soils with a variety of characteristics and promote the continuous supply (slow release) of nutrients to plants	(Cesari et al. 2020 ▶)
To prepare a dosage form in order to increase solubility of Naringin	Naringin loaded polycaprolactone microspheres.	Naringin loaded polycaprolactone microspheres for increased solubility of Naringin .	Solvent evaporation method	Three-level Box-Behnken configuration can be used to configure a Naringin-loaded polycaprolactone microspheres based oral suspension, demonstrating that Naringin solubility is greatly improved as evidenced by the optimized suspension's particle size	(Ghosal et al. 2018 ▶)
To increase water solubility, permeability and Bioavailability of Naringin	Naringin polymeric micelles	To make polymeric Naringin micelles based from pluronic F68 and test their antitumor activity in mice with Ehrlich ascites carcinoma	Thin film hydration technique	1:50 polymeric micelles containing PF68 may be a promising nanocarrier for the phytopharmaceutical Naringin, with increased water solubility, permeability, and bioavailability, and also increased antitumor and antiulcer activities	(Mohamed et al. 2018 ▶)

Ng-m-SAIB: Naringin-loaded microsphere/sucrose acetate isobutyrate; AuNPs: Gold nanoparticles; PCL: Polycaprolactone; Mw: Molecular weight; MCs: Microcapsules; PF68: Pluronic-F68

**Anti-depressant effect**

Naringin therapy may be helpful to achieve functional behavioral effects through enhancing the cholinergic transmission pathways (Ben-Azu et al., 2019 ▶). The post-stroke depression action of naringin showed that modulation of nitric oxide is involved in naringin's protective effect against post-stroke depression induced by bilateral common carotid artery occlusion (BCCAO) (Aggarwal et al., 2010 ▶). In another study, antipsychotic effects of naringin in ketamine induced deficits in rats showed that naringin has likely therapeutic add-on activity against schizophrenia induced by ketamine (George et al., 2020 ▶).

The summary of pharmacological activities is mentioned in Table 2.

Last few years have emerged as the era of development of novel formulation process technology. Novel drug delivery made it possible to overcome various flaws associated with the herbal formulations as well as plant isolates. So far great efforts have been made by researchers to develop various novel formulations of naringin or naringin-containing extracts. Various formulations are listed in Table 3.

## Discussion

Naringin, and other bioflavonoids, possess the properties to act as powerful antioxidants which have been often proven via *in vitro* experiments. Naringin, is a key component of flavonoid phytoconstituent, has the potential to assist in the treatment of a wide range of chronic degenerative diseases due to its diverse pharmacological properties. Further study is required to explain the activities of naringin inside the body and the rate and degree of absorption. The antioxidant potential of naringin metabolites and the pathways involved in metabolic translation need to be recognized and appraised to precisely determine the effect naringin *in vivo* and its efficiency in preventing diseases occurring due to oxidative damage. Low water solubility, less bioavailability and delayed release are major glitches to be worried. To overcome these questions various approaches are been tested such as formulation and evaluation of novel drug delivery form such as Nano formulations, liposomes, etc. These novel methods were utilized in improving the pharmacokinetic and pharmacological properties of naringin.

## Conflicts of interest

The authors have declared that there is no conflict of interest.
